# Mitral valve replacement in severe mitral annular calcification: Avoiding disaster

**DOI:** 10.1016/j.xjse.2024.100029

**Published:** 2024-09-27

**Authors:** Andreas Polycarpou, Matthew Soule, Rosemary Kelly

**Affiliations:** Division of Cardiothoracic Surgery, Department of Surgery, University of Minnesota, Minneapolis, Minn

**Figure:**
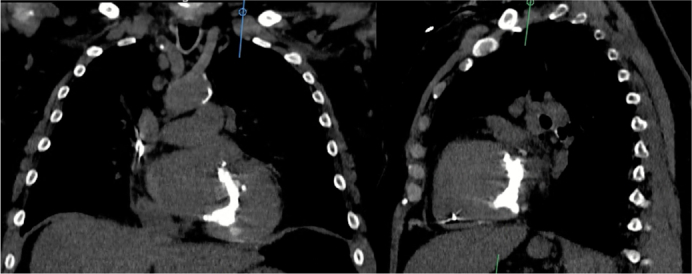
Chest computed tomography scan showing severe mitral annular calcification.

**Central Message**

Complex mitral annular debridement and decalcification can be done more safely and thoroughly using ultrasonic emulsification with a cavitronic ultrasonic surgical aspirator (CUSA).

Mitral annular calcification (MAC) significantly increases the technical complexity of mitral surgery, and its presence is a risk factor for perioperative complications and increased mortality. Here we describe our preferred surgical technique using ultrasonic emulsification to facilitate decalcification of severe MAC.

## Case Presentation

A 72-year-old female with known severe MAC presented with progressive dyspnea on exertion. Her comorbidities included diabetes mellitus, hypertension, atrial fibrillation with prior percutaneous ablation, sick sinus syndrome status-post permanent pacemaker implantation, and coronary artery disease with a history of percutaneous coronary intervention of the left anterior descending and circumflex arteries. Transthoracic echocardiography demonstrated severe mitral stenosis, with a mean gradient of 14 mm Hg, severe MAC, and mild mitral regurgitation. In addition, there was moderate-to-severe aortic stenosis and pulmonary arterial hypertension, with preserved biventricular function. Following a multidisciplinary heart team discussion and consideration of all treatment options, the patient was referred for surgical evaluation.

Preoperative coronary angiography demonstrated a right-dominant system, mild diffuse coronary artery disease, and patent stents (Videos 1 and 2). Severe MAC was readily evident on fluoroscopy. Right heart catheterization demonstrated elevated pulmonary arterial pressure at 60/38 mm Hg.

Preoperative transesophageal echocardiography (TEE) was obtained to better evaluate the relationship of the calcification, not only to the mitral annulus and mitral apparatus, but also to the aortic valve (AV) (Videos 3 and 4). TEE revealed calcification extending along the tissue of the mitral annulus into the papillary muscles. The mean gradient was 14 mm Hg across the mitral valve (MV) at a heart rate of 70 bpm and 23 mm Hg across the AV, with mild mitral regurgitation, mild aortic insufficiency, and trivial tricuspid regurgitation.

**Figure 1:**
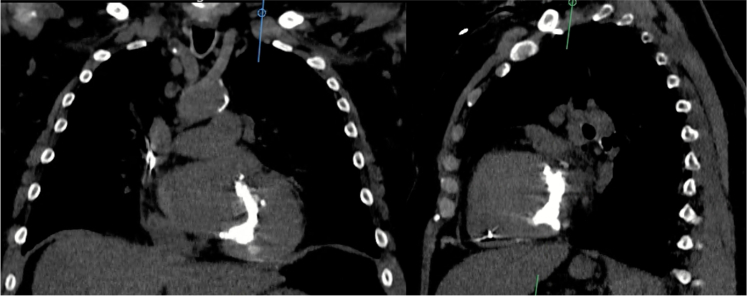
Chest computed tomography scan, coronal and sagittal views, demonstrating severe MAC.

As is our standard routine for MAC cases, a computed tomography scan was obtained for preoperative surgical planning. This scan confirmed nearly circumferential severe calcification of the mitral annulus, extending into the papillary muscles and the AV annulus (Figure 1).

## Operative Technique

**Figure 2:**
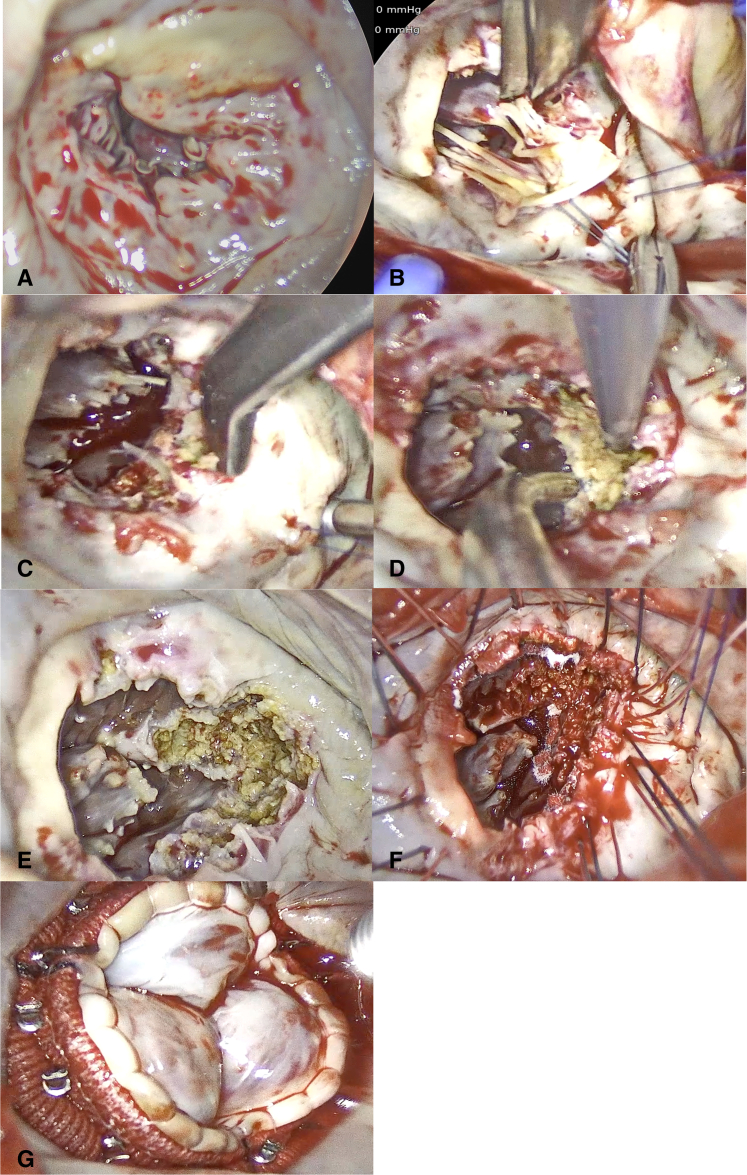
Key surgical steps. A, Mitral valve (*MV*) assessment. B, Anterior mitral leaflet resection. C, Manual debridement. D, Cavitronic ultrasonic surgical aspirator (*CUSA*) decalcification. E, Final result following manual debridement and CUSA decalcification. F, Placement of noninverting pledgeted mitral annular sutures. G, Seated bioprosthetic MV.

Surgery was offered, with a plan for primary sternotomy, MV replacement, AV replacement, a modified biatrial maze procedure, and left atrial appendage occlusion. Cardiopulmonary bypass was initiated, and a biatrial maze procedure was performed on the on-pump beating heart. Left atrial maze lesions were created using a bipolar radiofrequency ablation clamp, followed by right atrial maze using cryoablation. Subsequently, the aortic cross-clamp was applied, cardiac standstill was achieved, and an ascending aortotomy was performed. Non–coronary cusp aortic annular sutures were placed prior to exposing the MV, as we anticipated that tissue remaining in that region would be limited following mitral annular debridement. The MV was then exposed via a left atriotomy approach and assessed (Figure 2, *A*, Video 5). The MV was rigid and restricted, with nearly circumferential calcification. The MV had very limited mobility, and its orifice was approximately 1.5 cm in diameter.

We proceeded with resecting the anterior leaflet with its chordea, which allowed some access into the left ventricle (Figure 2, *B*, Video 5). This was followed by manual debridement using a rongeur and suction to debulk the calcification as much as feasible (Figure 2, *C*, Video 5). At this point, the mitral annulus was sized and was only able to accommodate a 25-mm MV prosthesis. Subsequently, a cavitronic ultrasonic surgical aspirator (CUSA) device was used to emulsify the remaining calcification embedded in the annulus (Figure 2, *D* and *E*, Video 5), allowing for its removal either manually or by suctioning while at the same time preserving the underlying tissue of the mitral annulus and the left ventricle to allow for reliable suture placement. To achieve optimal surgical results, it is advised that the surgeon slowly break up the calcification and carefully control the generated debris, with the aim of at least softening the annular calcium enough to allow for an appropriate-sized valve.

Following CUSA debridement, the mitral annulus was up-sized to a 29-mm bioprosthetic MV. Circumferential noneverting mitral annular sutures were placed in a ventricular-to-atrial direction using pledgeted double-armed 2-0 braided polyester suture on V7 needles, with pledgets on the ventricular side (Figure 2, *F*, Video 5), and a 29-mm mitral bioprosthesis was seated (Figure 2, *G*, Video 5). During annular suture placement, additional selective CUSA debridement was performed to further release the scarred annulus. The AV was then replaced with a 23-mm bioprosthesis, and the left atrial appendage was occluded using a clip device.

The patient had an uneventful postoperative course and was discharged to home on postoperative day 8, neurologically intact and in normal sinus rhythm. Postoperative transthoracic echocardiography demonstrated well-functioning prosthetic AV and MV without perivalvular leak, with a mean gradient of 5 mm Hg across the MV at a heart rate of 60 bpm and a mean gradient of 11 mm Hg across the AV.

## Discussion

MAC is a chronic degenerative process characterized by calcific alterations of the fibrous mitral annular skeleton that also may involve the left ventricular tissue, including the papillary muscles. Risk factors for MAC include advanced age, female sex, diabetes mellitus, renal failure, and metabolic calcium disorders. The presence of MAC not only increases operative mortality, but also heightens the risk for complications, such as atrioventricular groove disruption (AVGD), perivalvular leak, and heart block.^1^^,^^2^ Inadequate decalcification of MAC can lead to undersizing of the prosthetic MV, with resultant mitral stenosis.

No clear consensus exists as to the best surgical approach to MAC. Despite advancements with transcatheter interventions, to date, open cardiac surgery remains the gold standard in the management of MAC owing to the infeasibility of most transcatheter treatment options. Traditional surgical techniques involve manual annular debridement and patch reconstruction of the tenuous periannular tissues prior to implantation of the prosthetic MV.^3^ Our alternative technique for dealing with MAC using ultrasonic emulsification with CUSA has resulted in safe and effective valve replacements.

CUSA is a device traditionally used in liver surgery and is readily accessible in most modern operating theaters. The safety and efficacy of ultrasonic emulsification for MAC has been demonstrated in previous publications.^3^, ^4^, ^5^ CUSA generates high-frequency vibrations that induce cavitations in calcified tissues, leading to fragmentation and subsequent remodeling of the calcification without damaging adjacent soft tissues.^4^ The debris created in this process is aspirated simultaneously.

Operative “tips and tricks” of using CUSA for MAC include the following:

1. Minimizing manipulation and torque of the calcified mitral annulus to reduce the risk of AVGD
2. Ensuring decalcification of MAC while simultaneously preserving the left atrial and ventricular endocardium and muscle
3. Taking the time necessary for CUSA emulsification to ensure calcium remodeling, while avoiding injury to adjacent structures
4. Ensuring attentive assistance from the assistant surgeon with deliberate suctioning using an open-tip suction catheter to avoid embolization of debris.
5. Decalcifying “just enough” calcium to allow an appropriate-sized prosthetic valve.
6. Placing equally distributed, noneverting pledgeted annular sutures in a ventricle-to-atrium direction, taking care to incorporate the endocardium on both sides. This will help reinforce the mitral annulus and mitigate the risk for AVGD.
7. Our preferred CUSA settings: aspiration, 100%; irrigation, 8 to 10 mL/minute; amplitude, 80% to 100%; tissue select, 4+.

Given its ability to effectively remove calcium with the added safety of preserving adjacent healthy tissue, CUSA mitral annular decalcification is a useful surgical strategy that may help decrease the surgical risk of MAC.

## Conclusions

MAC poses a particular challenge to the cardiac surgeon performing MV surgery, and its presence increases the risk for catastrophic intraoperative and postoperative complications. Careful preoperative planning is of paramount importance to avoid disaster during MV surgery in the setting of MAC. Complex mitral annular debridement can be performed safely and effectively using CUSA emulsification, and we advocate for this additional tool to be part of every cardiac surgeon's armamentarium.

## Conflict of Interest Statement

The authors reported no conflicts of interest.

The *Journal* policy requires editors and reviewers to disclose conflicts of interest and to decline handling or reviewing manuscripts for which they may have a conflict of interest. The editors and reviewers of this article have no conflicts of interest.

## Supplementary Data

Video 1 Left coronary angiogram. Note the severe mitral annular calcification. Video available at: https://www.jtcvs.org/article/S2950-6050(24)00029-9/fulltext.

Video 2 Right coronary angiogram. Video available at: https://www.jtcvs.org/article/S2950-6050(24)00029-9/fulltext.

Video 3 Preoperative transesophageal echocardiography: 3-dimensional view of the severely stenotic mitral valve. Video available at: https://www.jtcvs.org/article/S2950-6050(24)00029-9/fulltext.

Video 4 Preoperative transesophageal echocardiography: color Doppler of the mitral valve. Video available at: https://www.jtcvs.org/article/S2950-6050(24)00029-9/fulltext.

Video 5 Key operative steps. Video available at: https://www.jtcvs.org/article/S2950-6050(24)00029-9/fulltext.
