# Supplementary figures and images for: Mitral valve replacement in severe mitral annular calcification: Avoiding disaster
Source: JTCVS Struct Endovasc. 2024 Sep 27;3:100029. doi: 10.1016/j.xjse.2024.100029 (PMC13244790; doi:10.1016/j.xjse.2024.100029)

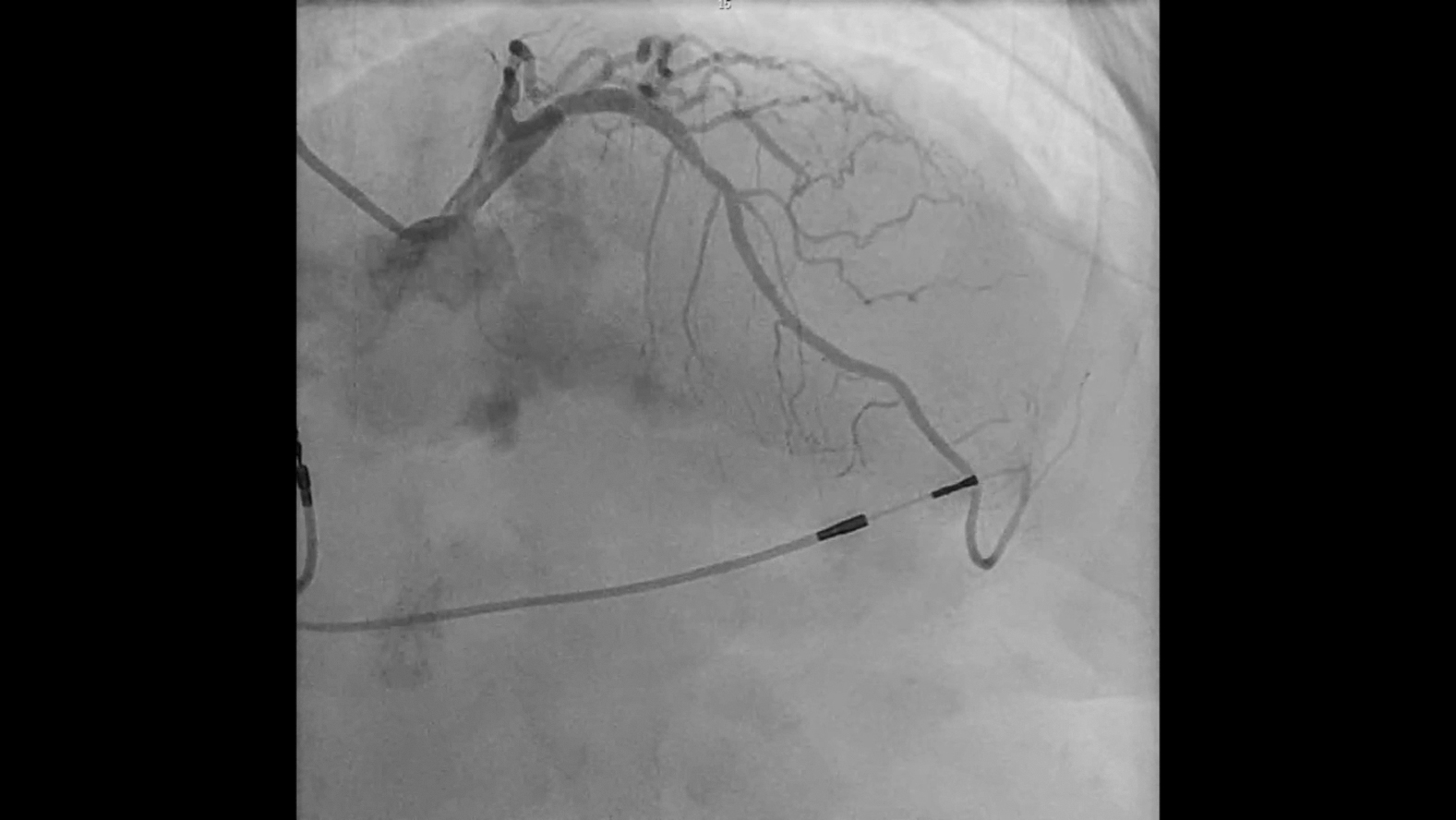

Supplement: Video 1 — Left coronary angiogram. Note the severe mitral annular calcification. Video available at: https://www.jtcvs.org/article/S2950-6050(24)00029-9/fulltext. [file fx2.jpg]

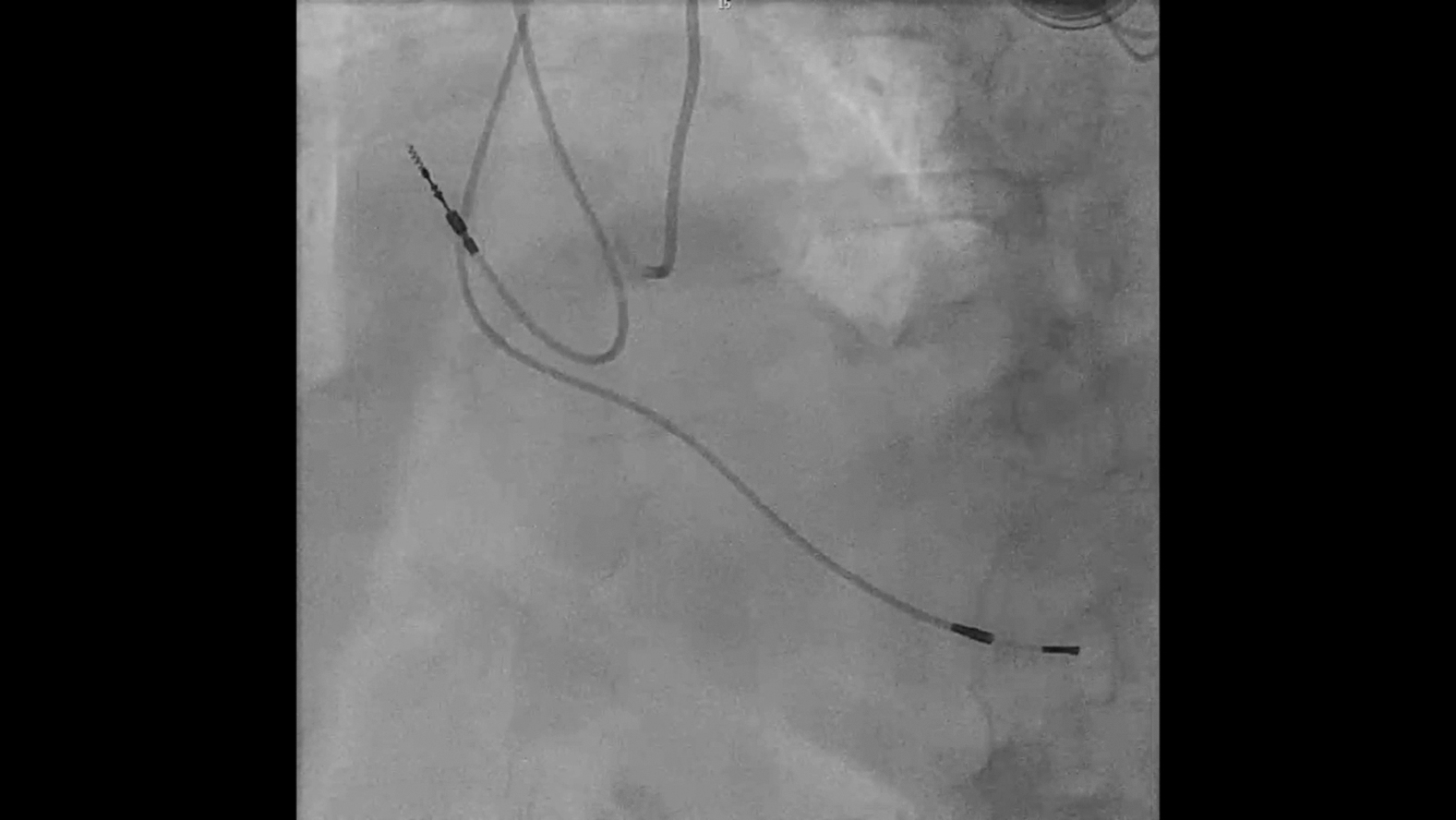

Supplement: Video 2 — Right coronary angiogram. Video available at: https://www.jtcvs.org/article/S2950-6050(24)00029-9/fulltext. [file fx3.jpg]

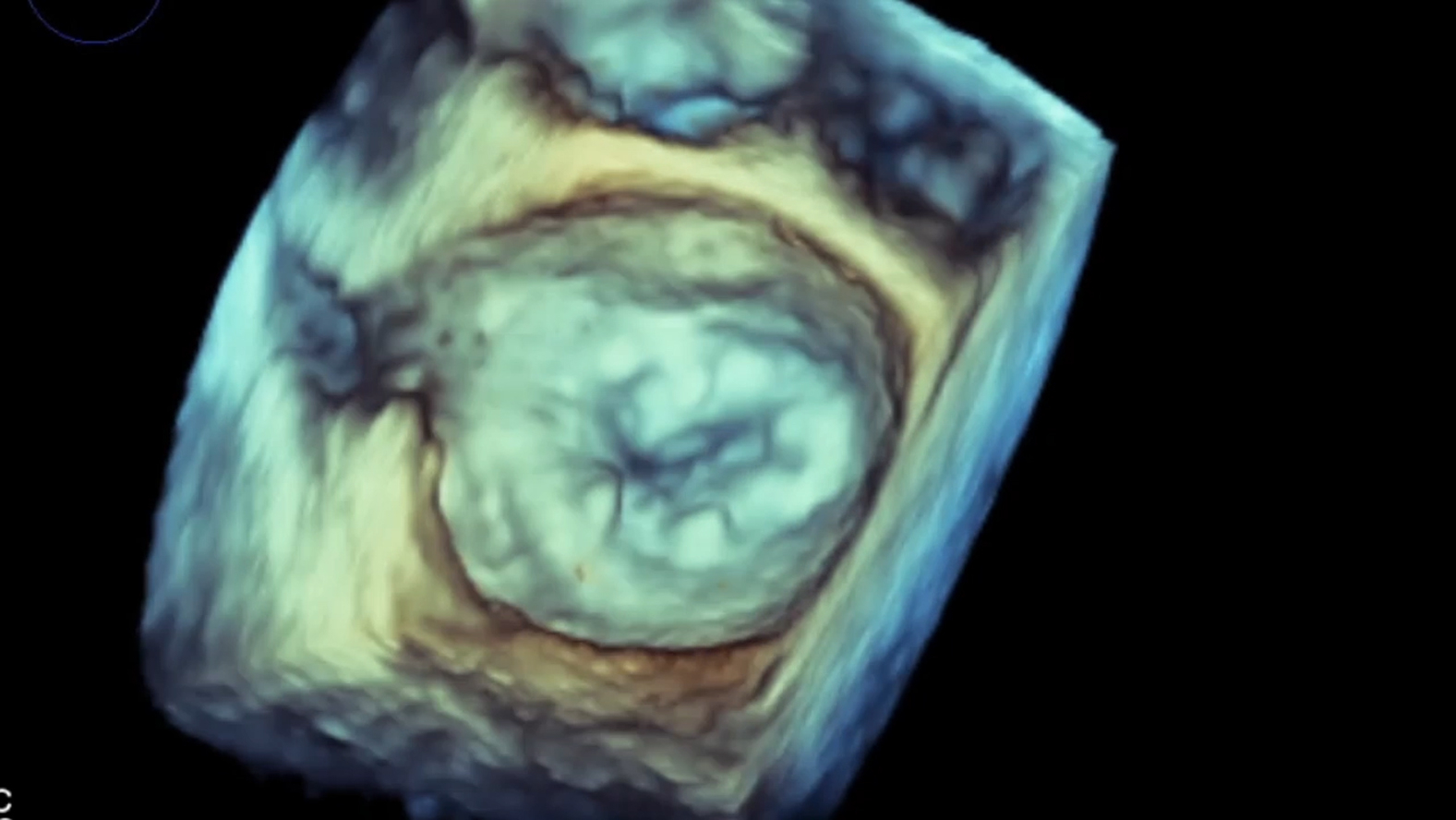

Supplement: Video 3 — Preoperative transesophageal echocardiography: 3-dimensional view of the severely stenotic mitral valve. Video available at: https://www.jtcvs.org/article/S2950-6050(24)00029-9/fulltext. [file fx4.jpg]

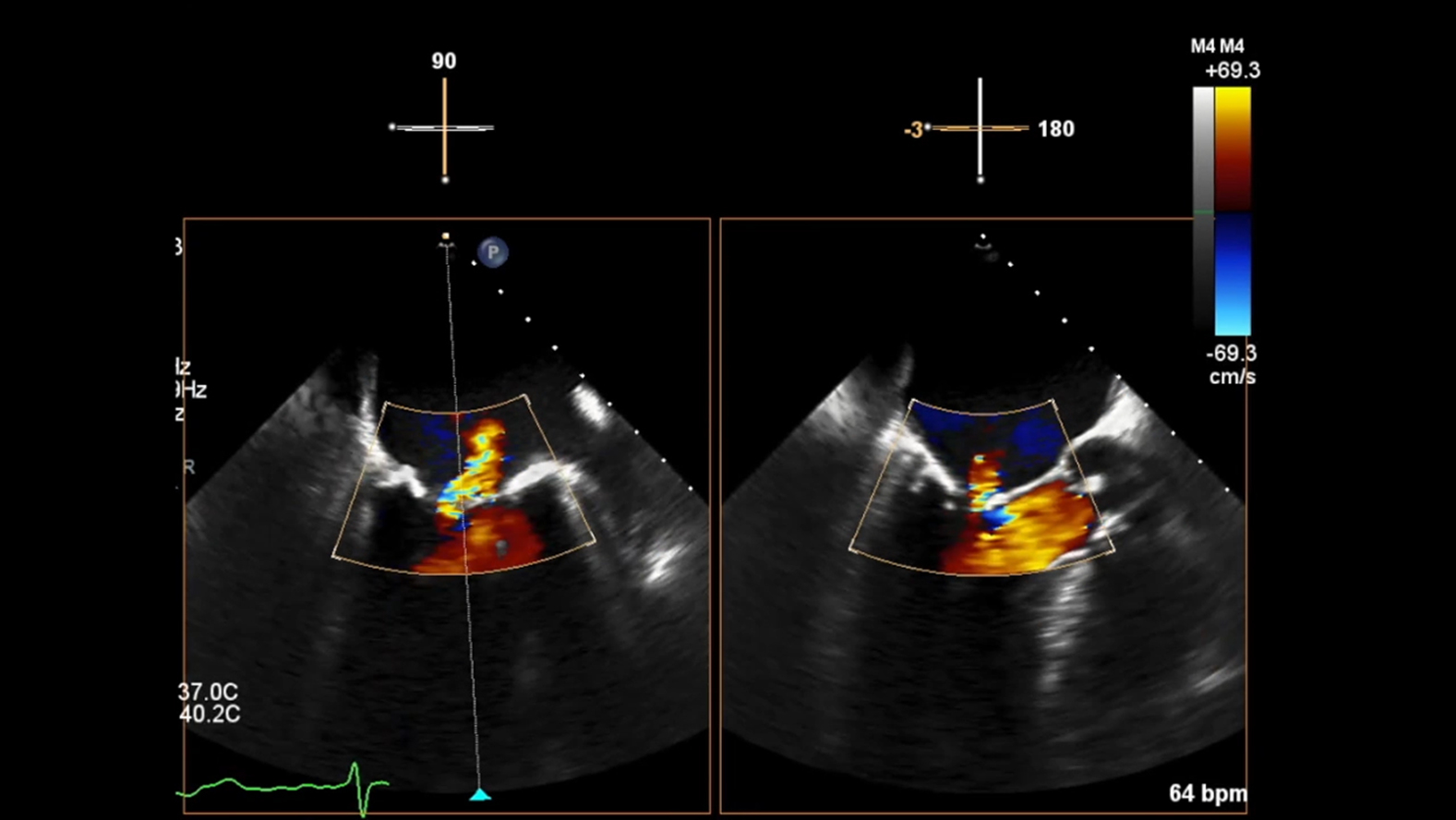

Supplement: Video 4 — Preoperative transesophageal echocardiography: color Doppler of the mitral valve. Video available at: https://www.jtcvs.org/article/S2950-6050(24)00029-9/fulltext. [file fx5.jpg]

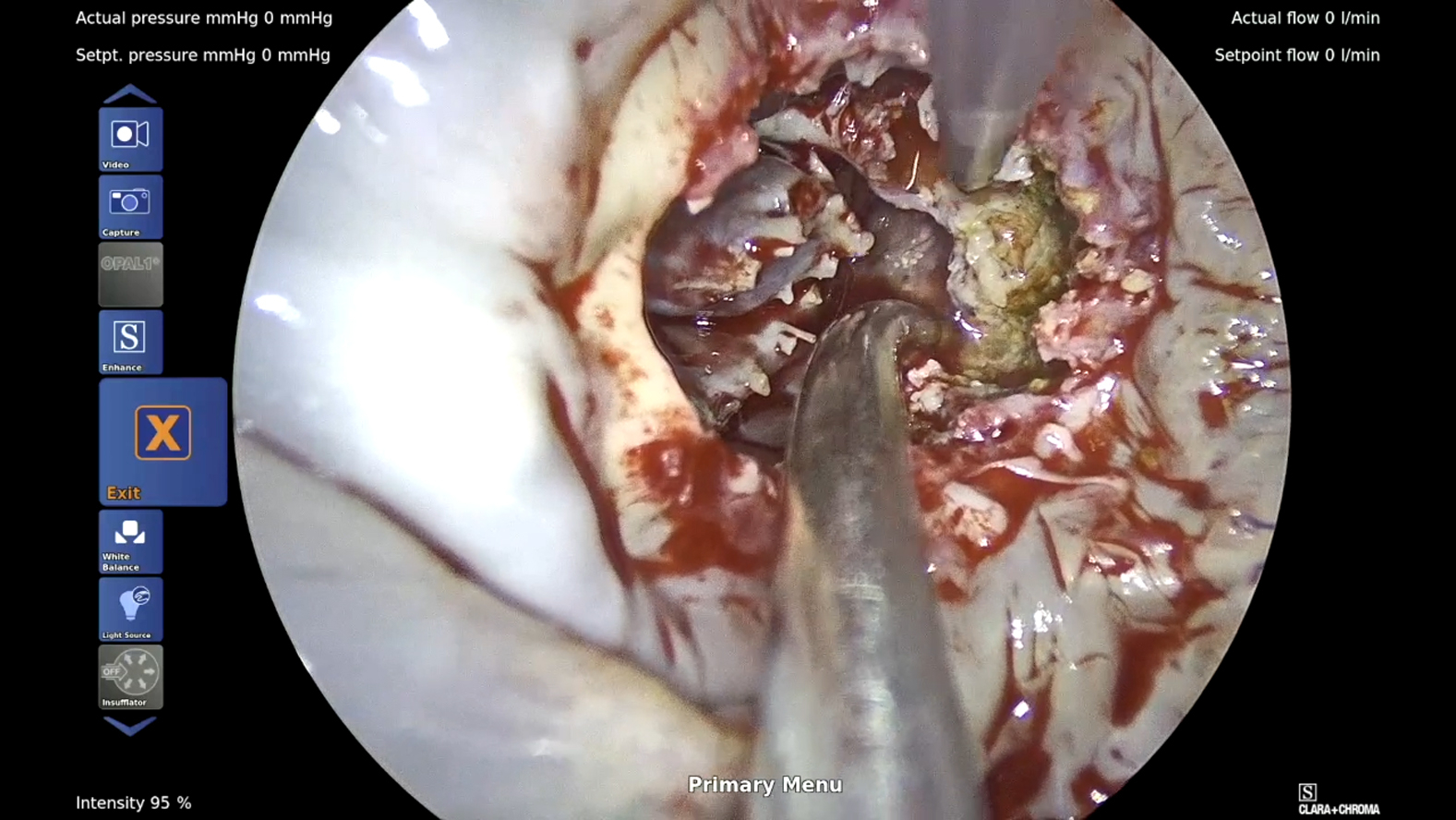

Supplement: Video 5 — Key operative steps. Video available at: https://www.jtcvs.org/article/S2950-6050(24)00029-9/fulltext. [file fx6.jpg]
